# Impact of remote ischemic postconditioning on acute ischemic stroke in China: a systematic review and meta-analysis of randomized controlled trials

**DOI:** 10.1186/s13643-024-02568-3

**Published:** 2024-05-30

**Authors:** Ming-Yuan Yan, Jin-Min Liu, Jing Wu, Qing Chang

**Affiliations:** 1 Beijing University of Chinese Medicine, Beijing, China; 2 Dongfang Hospital, Beijing University of Chinese Medicine, Beijing, 100078 China; 3 Dongzhimen Hospital, Beijing University of Chinese Medicine, Beijing, China

## Abstract

**Objective:**

Acute ischemic stroke (AIS) is a significant health burden in China, affecting a sizable portion of the population. Conventional pharmacological treatments frequently fall short of desirable outcomes. Therefore, exploring alternative therapies is crucial. Remote ischemic postconditioning (RIPostC) is a noninvasive and cost-effective adjunctive therapy. This study aimed to investigate the efficacy and safety of RIPostC as an adjunctive therapy for AIS to inform clinical practice.

**Methods:**

A comprehensive search was conducted across the PubMed, Embase, Web of Science, China National Knowledge Infrastructure (CNKI), WanFang, Weipu (VIP), and China Biology Medicine disc (CBM) databases up to October 2023. All included studies underwent bias risk assessment using the Cochrane risk-of-bias assessment tool. The primary outcome measure was the National Institute of Health Stroke Scale (NIHSS), with secondary outcomes including the Barthel index (BI), D-dimer, C-reactive protein (CRP), fibrinogen (FIB), brain-derived neurotrophic factor (BDNF), modified Rankin scale (mRS), interleukin-6 (IL-6), and tumor necrosis factor-α (TNF-α) levels. The data were analyzed using fixed-effects and random-effects models in Review Manager, with mean differences (MDs) and 95% confidence intervals (CIs) calculated for each outcome. The grading of recommendations, assessment, development, and evaluations (GRADE) approach was used to evaluate the level of evidence for each outcome measure.

**Results:**

This meta-analysis included 38 studies, encompassing 4334 patients. Compared with the control group, the RIPostC group had significantly lower NIHSS scores, serum CRP, D-dimer, IL-6, TNF-α, and FIB levels, and increased BDNF levels. Moreover, it improved the patient’s BI and mRS scores. According to the GRADE approach, the quality of evidence for mRS was deemed “moderate,” while the NIHSS, BI, and CRP were rated as “low” quality. IL-6, TNF-α, FIB, D-dimer, and BDNF received “very low” quality ratings.

**Conclusion:**

The findings suggest that RIPostC activates endogenous protective mechanisms, providing benefits to patients with AIS.

**Supplementary Information:**

The online version contains supplementary material available at 10.1186/s13643-024-02568-3.

## Introduction

Stroke is a significant neurological disorder characterized by high incidences of disability and mortality, ranking as the fifth leading cause of death in the United States and the primary cause in China [1–3]. Ischemic stroke, constituting approximately 70% of stroke cases in China [4], has a complex pathogenesis. Pathologies such as atherosclerosis and thrombosis in cerebral arteries may induce vascular spasms, stenosis, or occlusion, resulting in softening and necrosis of brain tissue and neurological deficits [5, 6]. Although intravenous thrombolysis is the primary treatment for acute cerebral infarction, its efficacy is limited by a narrow therapeutic window and contraindications associated with thrombolytic medications. Consequently, a considerable number of patients experience fatal outcomes or severe disabilities, significantly impacting their quality of life [4, 7]. Therefore, identifying effective strategies to counteract pathological alterations at the ischemic site and enhance recovery and quality of life is imperative. With the limited availability of exogenous methods to promote recovery from cerebral ischemia, exploring endogenous mechanisms for rehabilitation has become a focal point of interest.

Remote ischemic postconditioning (RIPostC) is an innovative and straightforward approach for safeguarding ischemic brain tissue. Its principle involves inducing brief, nonfatal ischemic episodes in noncritical organs following a life-threatening ischemic event in vital organs. This process triggers innate ischemic tolerance mechanisms, protecting the ischemic and damaged brain tissue [8]. RIPostC activates these endogenous protective mechanisms by subjecting specific organs or tissues to recurrent episodes of ischemia and reperfusion, which are insufficient to cause irreversible organ or tissue damage. However, these transient episodes stimulate the body’s internal protective response through RIPostC [9]. The precise mechanisms through which RIPostC exerts its protective effects are poorly understood; they are conventionally divided into fluid and immune-inflammatory regulatory mechanisms, with some interactions between them.

Regarding humoral regulation mechanisms, emerging research suggests that ischemic events prompt the body or tissues to produce anti-ischemic compounds or soluble substances, such as nitric oxide, adenosine, bradykinin, and vascular endothelial growth factor. These substances circulate in the bloodstream upon reperfusion, delivering bodily protective benefits. Moreover, RIPostC can stimulate local vascular endothelial cells to release cell vesicles containing proteins or microRNAs (miRNAs), which travel to the brain and modulate cellular activity, exerting a neuroprotective effect [10, 11].

Concerning immune-inflammatory regulatory mechanisms, both preclinical and clinical studies have indicated that RIPostC can inhibit proinflammatory responses, promote the transcription of anti-inflammatory and antiapoptotic genes, alleviate immune-inflammatory responses, and regulate peripheral immune cells, such as CD3+CD8+ T cells, B cells, CD3+/CD161a+ NKT cells, and anti-inflammatory CD43+/CD172a+ monocytes. Furthermore, it can adjust TNF-α and IL-6 levels to provide neuroprotection [12–14].

The primary evidence supporting the neuroprotective effects of RIPostC comes from animal studies, highlighting the involvement of various endogenous factors, including regulatory T cells, heat shock protein 70 (Hsp70), miRNAs, neuronal nitric oxide synthase, and brain-derived neurotrophic factor (BDNF) [15–20]. Clinical trials further support the potential of RIPostC to enhance recovery in patients with stroke, as evidenced by improved NIHSS scores, reduced high-sensitivity (hs)-CRP levels, diminished brain tissue infarct volume, and enhanced cognitive function post-stroke [21–25]. Theoretically, remote ischemic conditioning holds promise for facilitating disease recovery. However, the outcomes of a high-quality randomized controlled trial suggest that remote ischemic preconditioning may not significantly influence disease progression or recovery following acute ischemic stroke (AIS) [26]. Four systematic reviews and meta-analyses concerning RIPostC’s use in treating AIS have been examined; two focused on animal studies, potentially offering significant insights for researchers. The remaining two analyses pertained to clinical trials. However, these studies have relatively narrow outcome indicators, limiting a comprehensive understanding of RIPostC’s endogenous protective mechanisms. Consequently, this study was initiated to comprehensively assess the potential benefits of RIPostC in rehabilitating patients who have experienced AIS, incorporating a broader array of original research and outcome measures.

## Methods

### Systematic review protocol and registration

This review followed the Preferred Reporting Items for Systematic Reviews and Meta-Analyses (PRISMA) guidelines [27] and is registered with the International Prospective Register of Systematic Reviews (PROSPERO) (CRD42021261145).

### Search strategy

A comprehensive search was implemented in PubMed, Embase, Web of Science, China National Knowledge Infrastructure (CNKI), WanFang, Weipu (VIP), and China Biology Medicine disc (CBM) databases, extending the search timeframe up to April 2024. Keywords used in the search encompassed stroke, ischemic stroke, cerebral infarction, acute ischemic stroke, RIPostC, remote ischemic postconditioning, and randomized controlled trials, among others. The search strategy combined electronic database searches with manual efforts to ensure a thorough literature collection. The Additional file 1 details the search strategies (see Additional file 1).

#### Eligibility criteria

Inclusion criteria were as follows:

Diagnosis of AIS with significant symptoms, including unilateral facial or limb numbness, facial asymmetry, blurred vision, impaired visual rotation, balance issues, altered consciousness, convulsions, speech difficulties, hemiplegia, hemianopia, and hemisensory disorders
2) Head CT or MRI confirming the absence of cerebral hemorrhage
Patients aged between 18 and 90 years
Inclusion of the National Institute of Health Stroke Scale (NIHSS) among outcome indicators
Approval from the ethics committee of the conducting hospital
Utilization of a randomized controlled trial design
Comparison between a control group receiving intravenous thrombolysis or conventional drug therapy and an experimental group undergoing RIPostC alongside intravenous thrombolysis or conventional drug therapy
Research studies conducted in China

Exclusion criteria encompassed the following:

1. Duplicate entries, animal studies, review articles, conference abstracts, and case reports
2. Studies not aligning with the diagnosis of acute ischemic stroke, including transient ischemic attacks
3. Studies with incomplete outcome data that could not be extracted
4. Studies with intervention times and outcome indicators unsuitable for subgroup analysis with other studies
5. Inaccessibility of full texts
6. Outcome indicators not presented as mean ± standard deviation
7. Use of additional intervention measures alongside RIPostC and drug therapy
8. Studies conducted outside of China.
9. Non-randomized controlled trials (RCT)

#### Study selection and data extraction

**Fig. 1:**
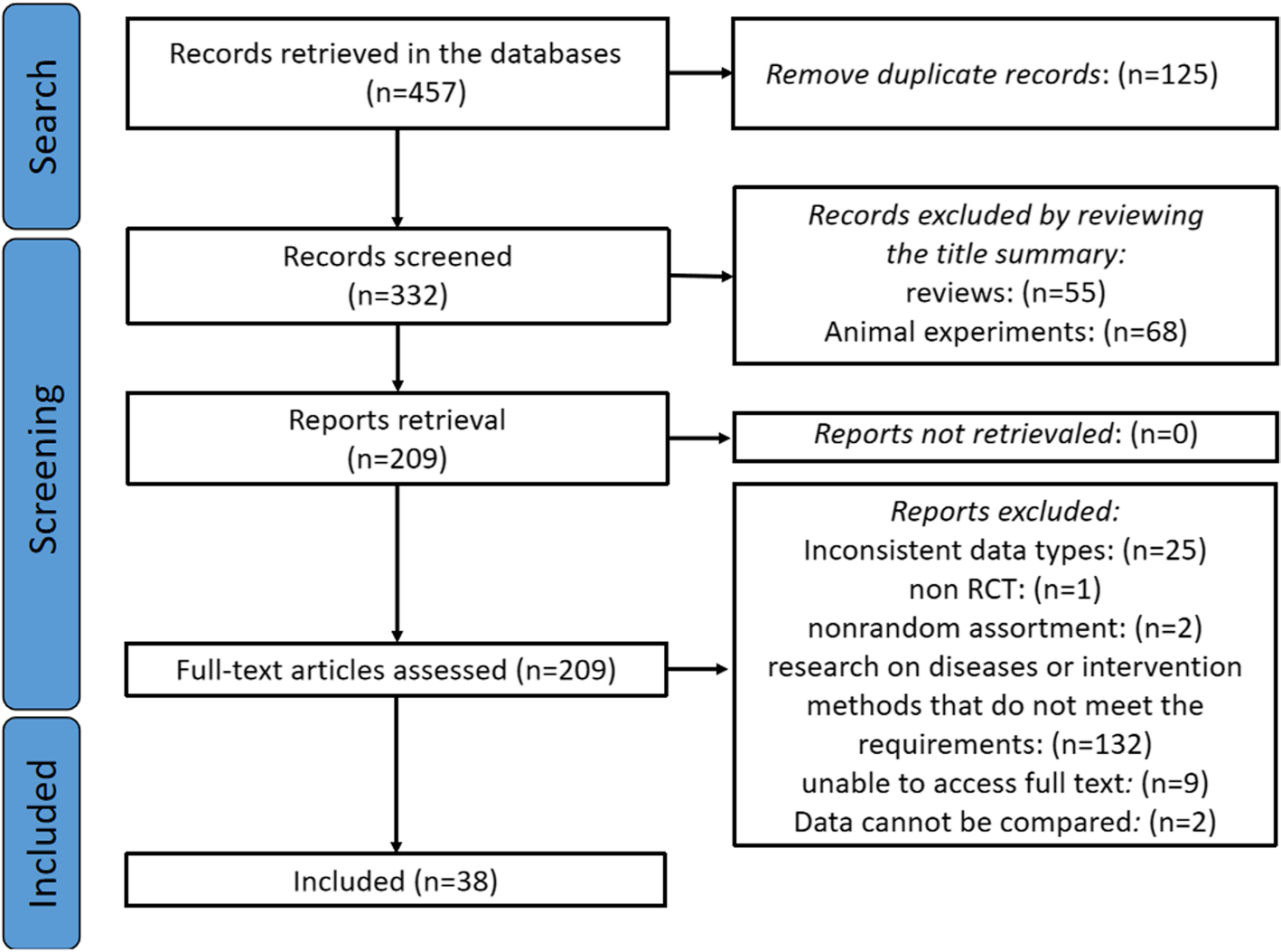
Flowchart illustrating the study screening process

Two researchers (M. Y. and J. W.) independently screened records for eligibility. After removing 125 duplicate records from the initial 457 retrieved records, 55 reviews and 68 animal experiments were excluded based on title and abstract. Full-text retrievals were attempted for the remaining records; however, nine were inaccessible. Full texts were reviewed for the remaining records, excluding 2 non-randomized studies, 1 non-RCT study, 132 studies not meeting the inclusion and exclusion criteria, and 25 studies with continuous variables not presented as mean ± standard deviation. Two records were excluded due to the inability to merge and analyze with any study at the intervention time point; the remaining 38 records met study requirements and were included. Disagreements were resolved by consulting a third reviewer (Q. C.), although the two researchers reached a unanimous agreement. Data extraction included first author, publication year, study design, participant demographics, interventions, control actions, duration of intervention, outcome measures, and adverse effects, with continuous data recorded as mean ± standard deviation in Excel sheets. The study screening methodology is illustrated in Fig. 1. The data extraction process is provided in the Additional files 2–5 (see Additional files 2–5).

### Statistical analysis

Statistical analyses were performed using Review Manager 5.3 (provided by the Cochrane Collaboration, Copenhagen, Denmark). The impact of RIPostC on continuous outcomes was assessed by calculating mean differences (MDs) with 95% confidence intervals (CI). The meta-analyses employed Mantel–Haenszel fixed-effects models in instances devoid of significant statistical heterogeneity among the studies. A significance threshold of 5% was maintained throughout the research. Heterogeneity among the studies was assessed using Cochran’s Q test and *I*^2^ methods. Acceptable homogeneity was defined as *P* > 0.1 and *I*^2^ < 50%, warranting a fixed-effects model; conversely, heterogeneity was assumed, prompting analysis via a random-effects model. The source of heterogeneity was explored through sensitivity analysis and subgroup examination.

### Target outcome indicators

The primary outcome measure was the National Institute of Health Stroke Scale (NIHSS), with additional outcome measures including the Barthel index (BI), D-dimer (D-D), C-reactive protein (CRP), fibrinogen (FIB), BDNF, modified Rankin scale (mRS), interleukin-6 (IL-6), and tumor necrosis factor-α (TNF-α).

## Results

### Study collection and characteristics

A total of 457 studies were retrieved, and after following the screening process, 38 randomized controlled trials [4, 28–64] conducted in China were selected for this meta-analysis. These studies, published between 2012 and 2023, involved 4324 participants, with 2166 in the control group and 2158 in the experimental group. All included studies were categorized based on the duration of RIPostC implementation to enhance the reliability of our data analysis. This categorization included a 3-day group with 3 studies [59–61], a 7-day group comprising 9 studies [29, 35–37, 41, 42, 47, 58, 64], a 10-day group with 3 studies [44–46], a 14-day group consisting of 15 studies [4, 28, 31, 40, 48–53, 55–57, 60, 61], a 3-month group with 2 studies [30, 38], and a 6-month group encompassing 6 studies [32–34, 39, 43, 54]. Detailed information and characteristics of the studies are listed in Tables 1 and 2.

**Table 1:** Basic characteristics of the included studies

**Study**	**Sample size**		**Age (years)**	**Male/female**		**Population**	**Interventions**
	**E**	**C**	**E / C**	**E**	**C**		**E / C**
Chen et al. [28]	36	36	61.55 ± 8.53/61.78 ± 8.63	23/13	22/14	Acute ischemic stroke within 24 h	RIPostC + routine treatment/routine treatment
Fan et al. [29]	32	32	61.33 ± 9.21/62.15 ± 8.32	19/13	17/15	Acute ischemic stroke within 6 h	RIPostC + thrombolysis/thrombolysis
Huang et al. [30]	80	80	61.8 ± 9.9/57.7 ± 9.6	45/35	41/39	Acute ischemic stroke 24–72 h	RIPostC + routine treatment/routine treatment
Yao et al. [31]	75	75	55.8 ± 8.63/54.89 ± 8.39	39/36	41/43	Acute ischemic stroke	RIPostC + routine treatment/routine treatment
Liu et al. [32]	38	38	54.2 ± 4.2/54.5 ± 4.1	23/15	25/13	Acute ischemic stroke	RIPostC + routine treatment/routine treatment
Jiang et al. [33]	41	41	64.82 ± 8.55/65.31 ± 7.36	22/19	25/16	Acute ischemic stroke within 48 h	RIPostC + routine treatment/routine treatment
Zhou et al. [34]	144	127	60.1 ± 19.3/57.9 ± 20.1	97/47	90/37	Acute ischemic stroke within 48 h	RIPostC + routine treatment/routine treatment
Kuang et al. [35]	31	30	Not report	Not report		Acute ischemic stroke	RIPostC + routine treatment/routine treatment
Fang et al. [36]	28	30	63.68 ± 8.61/61.60 ± 11.95	16/12	17/13	Acute ischemic stroke 24–72 h	RIPostC + routine treatment/routine treatment
Mai et al. [37]	30	30	63.7 ± 13.5/68.7 ± 11.5	16/14	20/10	Acute ischemic stroke within 72 h	RIPostC + routine treatment/routine treatment
Zhang et al. [38]	60	60	65.61 ± 5.46/65.46 ± 5.84	35/25	33/27	Acute ischemic stroke within 24 h	RIPostC + routine treatment/routine treatment
Liu et al. [39]	52	52	60.1 ± 4.8/60.7 ± 5.0	30/22	32/20	Acute ischemic stroke within 48 h	RIPostC + routine treatment/routine treatment
Li et al. [40]	119	120	63.15 ± 10.12/64.64 ± 9.63	75/44	85/35	Acute ischemic stroke within 72 h	RIPostC + routine treatment/routine treatment
Li et al. [41]	23	27	59.8 ± 8.96/61.34 ± 9.64	13/10	15/12	Acute ischemic stroke within 48 h	RIPostC + routine treatment/routine treatment
Chen et al. [42]	15	15	61.53 ± 9.70/59.13 ± 12.51	9/6	8/7	Acute ischemic stroke	RIPostC + thrombolysis/thrombolysis
Lin et al. [43]	40	40	65.37 ± 5.16/67.25 ± 6.32	22/18	24/16	Acute ischemic stroke within 48 h	RIPostC + routine treatment/routine treatment
Wu et al. [44]	50	50	65.37 ± 5.16/67.25 ± 6.32	23/27	24/26	Acute ischemic stroke within 24 h	RIPostC + routine treatment/routine treatment
Liu et al. [45]	29	29	67.52 ± 7.71/62.72 ± 11.09	17/12	16/13	Acute ischemic stroke within 24 h	RIPostC + routine treatment/routine treatment
Chen et al. [46]	50	50	60.32 ± 10.53/60.43 ± 11.22	27/23	22/28	Acute ischemic stroke within 72 h	RIPostC + routine treatment/routine treatment
Zhou et al. [47]	50	50	61.86 ± 8.27/62.54 ± 8.49	27/23	28/22	Acute ischemic stroke within 72 h	RIPostC + routine treatment/routine treatment
Li et al. [48]	113	114	65.60 ± 5.20/66.20 ± 6.40	72/41	82/32	Acute ischemic stroke within 72 h	RIPostC + routine treatment/routine treatment
Dai et al. [49]	70	70	64.16 ± 8.91/63.77 ± 7.71	38/32	40/30	Acute ischemic stroke within 24 h	RIPostC + routine treatment/routine treatment
Wu et al. [50]	70	70	64.16 ± 8.91/63.77 ± 7.71	38/32	40/30	Acute ischemic stroke within 24 h	RIPostC + routine treatment/routine treatment
Wang et al. [51]	70	70	64.16 ± 8.91/63.77 ± 7.71	38/32	40/30	Acute ischemic stroke within 24 h	RIPostC + routine treatment/routine treatment
Qu et al. [52]	45	44	63.51 ± 9.34/61.80 ± 9.54	24/21	22/22	Acute ischemic stroke 6 h-72 h	RIPostC + routine treatment/routine treatment
Sun et al. [53]	47	47	55.18 ± 5.65/54.87 ± 5.37	30/17	28/19	Acute ischemic stroke within 4.5 h	RIPostC + thrombolysis/thrombolysis
Feng et al. [54]	75	55	67.2 ± 3.1/67.0 ± 3.3	49/26	35/20	Acute ischemic stroke within 48 h	RIPostC + routine treatment/routine treatment
Guo et al. [55]	48	48	68.04 ± 4.61/67.89 ± 4.55	26/22	25/23	Acute ischemic stroke within 4 h	RIPostC + thrombolysis/thrombolysis
Zhao et al. [56]	59	57	64.16 ± 8.91/63.77 ± 7.71	37/22	35/24	Acute ischemic stroke within 72 h	RIPostC + routine treatment/routine treatment
Peng et al. [4]	20	20	Not report	Not report		Acute ischemic stroke within 72 h	RIPostC + routine treatment/routine treatment
Meng et al. [57]	69	69	Not report	Not report		Acute ischemic stroke 6–72 h	RIPostC + routine treatment/routine treatment
Shi et al. [58]	74	62	60.5 ± 3.8/61.5 ± 4.5	47/27	45/17	Acute ischemic stroke within 72 h	RIPostC + routine treatment/routine treatment
Wang et al. [59]	145	145	Not report	Not report		Acute ischemic stroke within 48 h	RIPostC + routine treatment/routine treatment
Tian et al. [60]	18	18	57.5 ± 2.6/57.7 ± 2.7	12/6	11/7	Acute ischemic stroke	RIPostC + routine treatment/routine treatment
Wang et al. [61]	9	9	Not report	5/4	4/5	Acute ischemic stroke	RIPostC + routine treatment/routine treatment
Wu et al. [62]	60	60	Not report	Not report		Acute ischemic stroke within 7 days	RIPostC + routine treatment/routine treatment
Zhang et al. [63]	150	136	55.8 ± 8.63/54.89 ± 8.39	84/66	69/67	Acute ischemic stroke within 72 h	RIPostC + routine treatment/routine treatment
Zhao et al. [64]	66	65	59.67 ± 11.54/58.58 ± 10.97	31/35	29/36	Acute ischemic stroke within 72 h	RIPostC + routine treatment/routine treatment

**Table 2:** Specific information about the included randomized controlled trials

**Study**	**RIPostC design**					**Treatment methods for the control groups**
	Cycles	Ischemia time/ reperfusion time	Operate pressure	Operated limb	Treatment duration	
Chen et al. [28]	5	5 min/5 min	200–220 mmHg	Upper arm	14 days	Oxygen inhalation, aspirin, edaravone, TCM: Xuesaitong, adjusting blood sugar, blood pressure, and blood lipids
Fan et al. [29]	3	5 min/5 min	200 mmHg	Upper arm	7 days	Intraveneuze trombolyse, anticoagulant, antiplatelet, blood lipid regulation, elimination of oxygen free radicals, brain protection
Huang et al. [30]	5	5 min/5 min	200 mmHg	Upper arm	90 days	Antiplatelet aggregation, lowering blood lipids, stabilizing plaques, stabilizing blood pressure, improving circulation, and dilating blood vessels
Yao et al. [31]	5	5 min/5 min	180–200 mmHg	Upper arm	7–14 days	Based on guidelines, conventional antiplatelet and oral statin therapy are administered
Liu et al. [32]	5	5 min/5 min	190 mmHg	Upper arm	180 days	Treatment for improving coagulation function, lowering blood lipids, lowering blood pressure, nourishing nerves, etc.
Jiang et al. [33]	10	5 min/5 min	200 mmHg	Upper arm	180 days	Conventional treatment medication to control risk factors such as blood sugar, blood lipids, and hypertension at normal levels
Zhou et al. [34]	5	5 min/5 min	180–200 mmHg	Upper arm	28 days	Purely using medication for treatment, mainly for antiplatelet aggregation, promoting blood circulation and removing blood stasis, etc.
Kuang et al. [35]	3	5 min/5 min	200 mmHg	Upper arm	7 days	Antiplatelet aggregation, stabilizing plaques, promoting blood circulation and removing blood stasis, clearing oxygen-free radicals
Fang et al. [36]	3	5 min/5 min	Not report	Upper arm	7 days	Antiplatelet aggregation and lipid-lowering stabilization of plaques, clearing free radicals, improving circulation, controlling blood pressure and blood sugar, etc.
Mai et al. [37]	4	5 min/5 min	Not report	Upper arm	7 days	Antiplatelet aggregation, lipid-lowering stabilization of plaques, improvement of circulation, oxygen uptake, etc.
Zhang et al. [38]	10	5 min/5 min	27 kPa	Upper arm	90 days	Routine interventions such as basic medication for cerebral infarction, fluid supplementation, maintenance of acid-base and electrolyte balance, and low-cholesterol diet
Liu et al. [39]	5	5 min/5 min	180–200 mmHg	Upper arm	180 days	Conventional drug therapy, including antiplatelet aggregation, blood circulation, and stasis elimination
Li et al. [40]	5	5 min/5 min	200 mmHg	Upper arm	14 days	Aspirin, atorvastatin, edaravone, butylphthalide, etc.
Li et al. [41]	4	5 min/5 min	200 mmHg	Upper arm	7 days	Routine treatment for cerebral infarction and left upper limb 60-mmHg compression
Chen et al. [42]	3	5 min/5 min	200 mmHg	Upper arm	7 days	Intraveneuze trombolyse
Lin et al. [43]	10	5 min/5 min	200 mmHg	Upper arm	180 days	Refer to the 2018 Chinese Guidelines for the Diagnosis and Treatment of Acute Ischemic Stroke to receive the best secondary preventive treatment
Wu et al. [44]	5	5 min/5 min	180 mmHg	Upper arm	10 days	Antiplatelet aggregation, lipid-lowering and stabilizing plaques, clearing free radicals, promoting blood circulation and removing blood stasis, nourishing nerves, lowering blood pressure, lowering blood sugar, etc.
Liu et al. [45]	5	5 min/5 min	180 mmHg	Upper arm	10 days	Routine neurology treatment
Chen et al. [46]	5	5 min/5 min	180 mmHg	Upper arm	10 days	TCM: Xuesaitong, Olaxitan, antiplatelet, agglomeration, basic disease treatment
Zhou et al. [47]	5	5 min/5 min	200 mmHg	Upper arm	7 days	Atorvastatin, clopidogrel, aspirin
Li et al. [48]	5	5 min/5 min	200 mmHg	Upper arm	14 days	All patients were treated symptomatically according to the 2018 Chinese Guidelines for the Diagnosis and Treatment of Acute Ischemic Stroke
Dai et al. [49]	5	5 min/5 min	200 mmHg	Upper arm	14 days	Improve cerebral blood supply, nourish nerves, resist platelet aggregation, lower intracranial pressure, lower lipid levels, and stabilize plaques
Wu et al. [50]	5	5 min/5 min	200 mmHg	Upper arm	14 days	Improving cerebral circulation, nourishing brain cells, lowering intracranial pressure, antiplatelet aggregation, lipid lowering, and stabilizing plaques
Wang et al. [51]	5	5 min/5 min	200 mmHg	Upper arm	14 days	According to the 2018 Chinese Guidelines for the Diagnosis and Treatment of Acute Ischemic Stroke, basic medication with conventional standards is administered
Qu et al. [52]	5	5 min/5 min	Not report	Upper arm	14 days	Standard treatment plan for acute cerebral infarction
Sun et al. [53]	4	3 min/3 min	200 mmHg	Upper arm	14 days	Intravenous thrombolysis, aspirin, mannitol
Feng et al. [54]	5	5 min/10 min	180–200 mmHg	Upper arm	180 days	Antiplatelet, anticoagulant, antihypertensive and lipid lowering, nutritional nerve therapy
Guo et al. [55]	3–4	5 min/5 min	200 mmHg	Upper arm	14 days	Vascular dilation, oxygen inhalation, intravenous thrombolysis
Zhao et al. [56]	5	5 min/5 min	200 mmHg	Upper arm	14 days	Aspirin, atorvastatin, edaravone, and butylphthalide
Peng et al. [4]	3	5 min/10 min	Not report	Thigh	14 days	Administer appropriate dehydrating agents according to the condition, control blood pressure and blood sugar, use drugs that promote blood circulation, remove blood stasis, and unblock collaterals, as well as anticoagulants Antiplatelet agents and symptomatic support, etc.
Meng et al. [57]	3	5 min/5 min	200 mmHg	Upper arm	3 days	Inhibiting platelet aggregation, clearing free radicals, protecting brain cells, improving cerebral circulation, lowering intracranial pressure, controlling blood pressure, blood glucose, and lipids, as well as preventing and treating various complications such as electrolyte disorders and infections, to support treatment
Shi et al. [58]	4	5 min/5 min	Not report	Upper arm	7 days	Aspirin, atorvastatin, clearing free radicals, controlling blood sugar, blood pressure, nourishing nerves, maintaining water electrolyte balance, etc.
Wang et al. [59]	3	5 min/5 min	200mmHg	Upper arm	3 days	Clearing free radicals, promoting blood circulation and removing blood stasis, improving circulation and protecting the brain, etc.
Tian et al. [60]	3	5 min/not report	200 mmHg	Upper arm	14 days	Inhibiting platelet aggregation, improving cerebral circulation, lowering intracranial pressure, lowering blood sugar, etc.
Wang et al. [61]	3	5 min/5 min	200 mmHg	Upper arm	14 days	Routine neurological treatment for cerebral infarction
Wu et al. [62]	3	5 min/5 min	200 mmHg	Upper arm	3 days	Routine neurological treatment for cerebral infarction
Zhang et al. [63]	3	5 min/5 min	200 mmHg	Upper arm	3 days	Antiplatelet aggregation, elimination of free radicals, brain protection, improvement of circulation, etc.
Zhao et al. [64]	10	5 min/5 min	200 mmHg	Upper arm	7 days	Aspirin, atorvastatin, edaravone, TCM: Xuesaitong, fluid replacement, correction of electrolyte disorders, and symptomatic supportive treatment

*TCM*, traditional Chinese medicine; *RT*, routine treatment; 1 mmHg = 0.133 kPa

### Outcome measurements

#### The National Institutes of Health Stroke Scale (NIHSS)

All included studies reported NIHSS scores. For a thorough meta-analysis, these studies were categorized based on the NIHSS measurement timelines: 3-day, 7-day, 10-day, 14-day, and 6-month groups. A higher NIHSS score indicates more severe nerve damage in the patient, while a lower score indicates better recovery of damaged nerves.

#### 3-day group

**Fig. 2:**
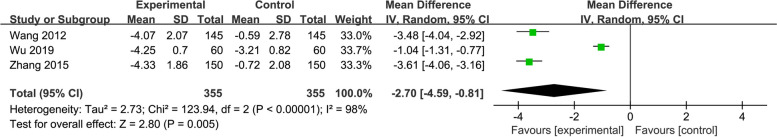
Forest plot of NIHSS in 3-day group

This group comprised 710 participants [59–61], with 355 each in the experimental and control groups. The outcomes at 14-day post-treatment were collectively reported; hence, the analysis focused on these results. The meta-analysis showed that RIPostC significantly lowered NIHSS scores compared to the control group, employing a random-effects model (*MD*: −2.70; 95% *CI*: −4.95, −0.81; *P* = 0.005). However, substantial heterogeneity across these trials was observed (*χ*^2^ = 123.94; *I*^2^ = 98%; *P* < 0.00001; Fig. 2).

#### 7-day group

**Fig. 3:**
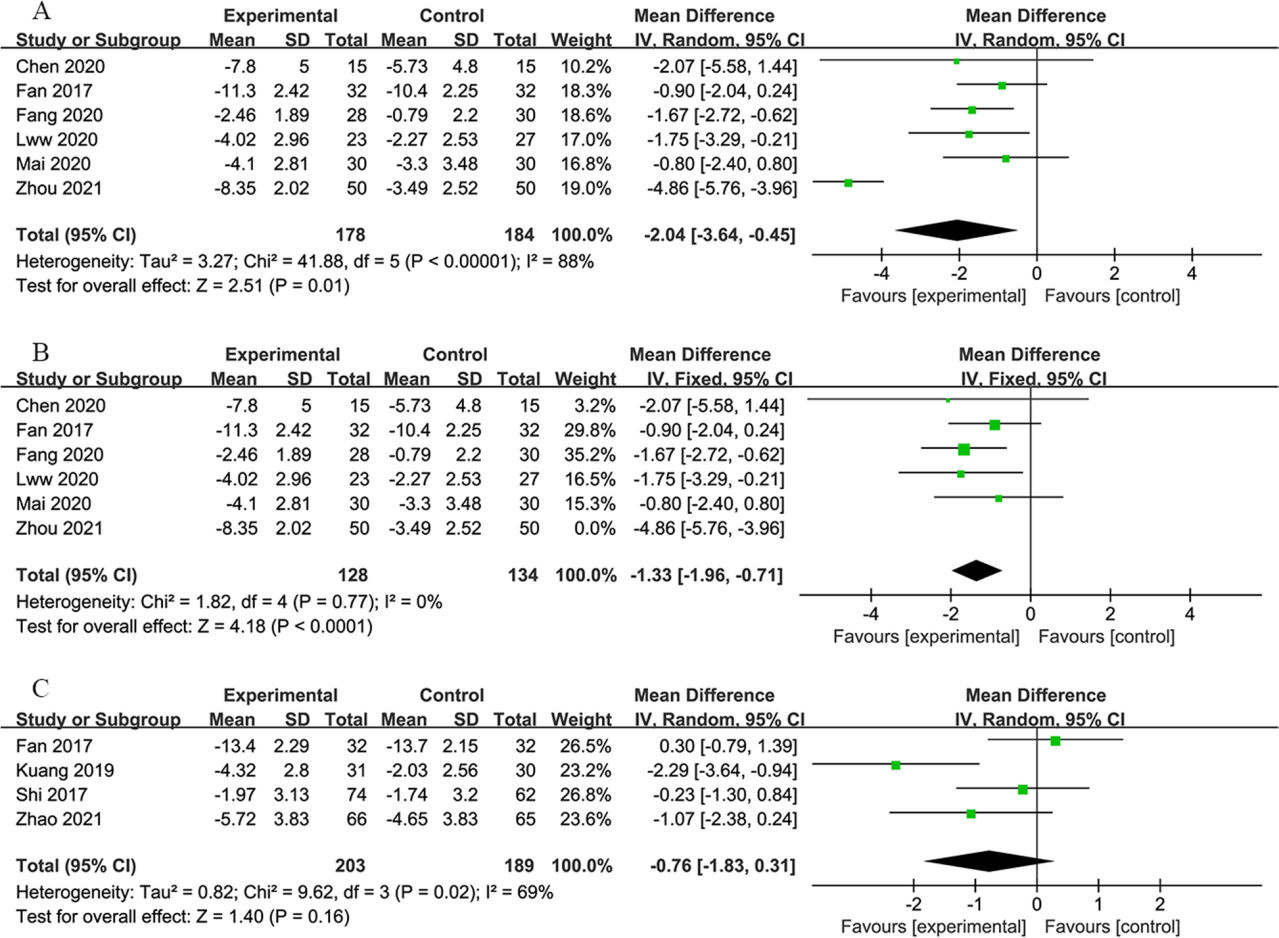
**A** Forest plot of NIHSS on 7th day in 7-day group. **B** Forest plot of NIHSS on 7th day in 7-day group without Zhou et al.’s study. **C** Forest plot of NIHSS on 14th day in 7-day group

This cohort included 349 participants in the experimental group and 341 in the control group, with outcomes reported on both the 7th day (across 6 studies [29, 36, 37, 41, 42, 47]) and the 14th day (across four studies [29, 35, 58, 64]) of treatment. The meta-analysis indicated a significant reduction in NIHSS scores by RIPostC on the 7th day compared to the control group, as determined through a random-effects model (*MD*: −2.04; 95% *CI*: −3.64, −0.45; *P* = 0.01; Fig. 3A). Significant heterogeneity was observed among the included trials. A subsequent sensitivity analysis revealed that the study by Zhou et al. [47] significantly influenced this heterogeneity. Upon its exclusion, heterogeneity was no longer present (*MD*: −1.33; 95% *CI*: −1.96, −0.71; *P* < 0.0001; Fig. 3B). Similarly, by the 14th-day post-treatment, RIPostC was found to reduce the NIHSS scores compared to the control group (*MD*: −0.76; 95% *CI*: −1.83, 0.31; *P* = 0.16; Fig. 3C).

#### 10-day group

**Fig. 4:**
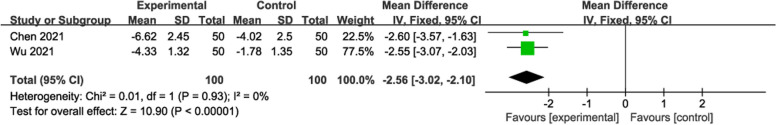
Forest plot of NIHSS in 10-day group

This group comprised 100 participants in the experimental group and 100 in the control group, with results reported on the 10th day of treatment [44, 46]. Compared to the control group, the experimental group exhibited a significant reduction in NIHSS scores, as depicted in Fig. 4, using a fixed-effects model (*MD*: −2.56; 95% *CI*: −3.02, −2.10; *P* < 0.00001).

#### 14-day group

**Fig. 5:**
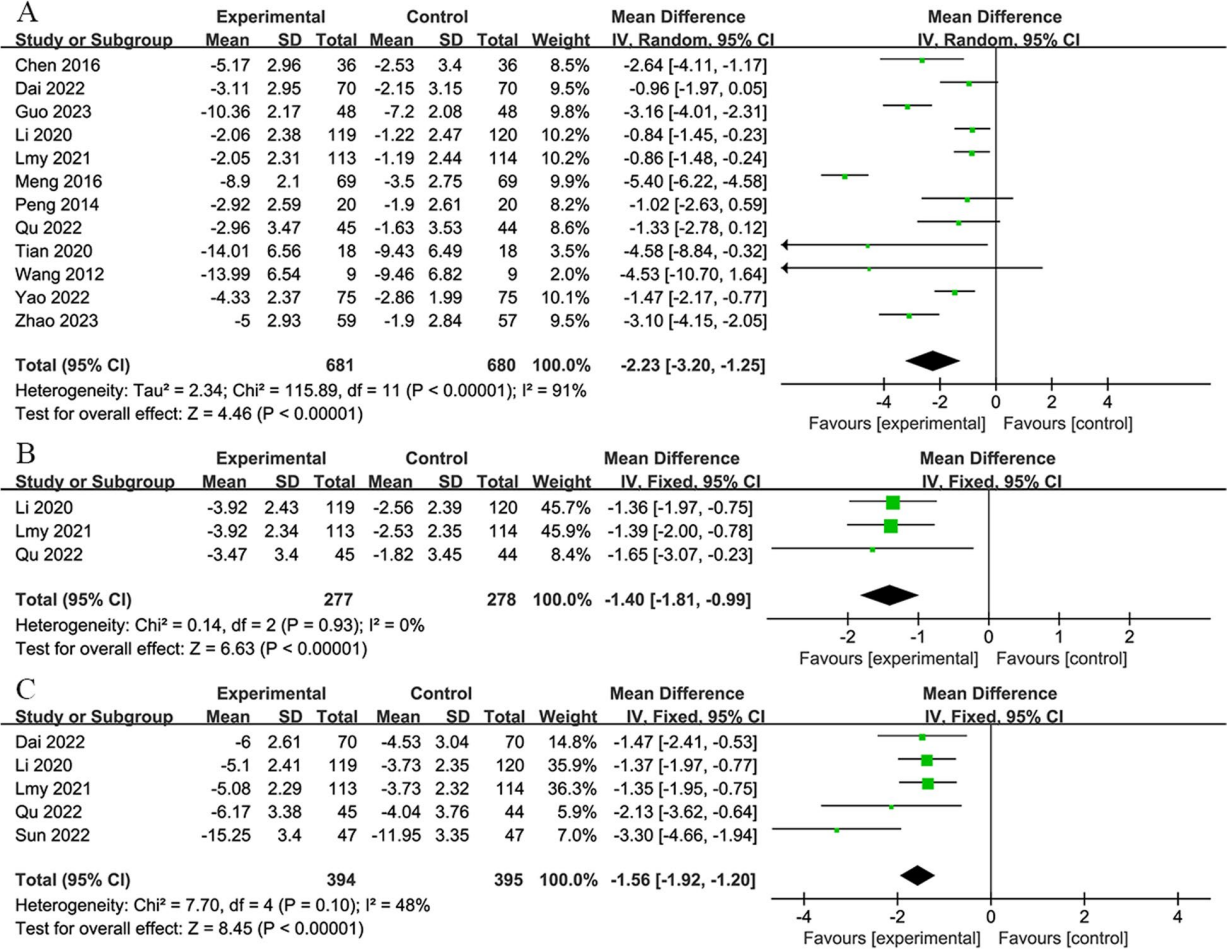
**A** Forest plot of NIHSS on 14th day in 14-day group. **B** Forest plot of NIHSS on 30th day in 14-day group. **C** Forest plot of NIHSS on 90th day in 14-day group

This group comprised 15 studies, with 938 participants in the experimental group and 937 in the control group. Twelve studies [4, 28, 31, 39, 48, 49, 52, 55–57, 60, 61] reported results on day 14 using a random-effects model (*MD*: −2.23; 95% *CI*: −3.20, −1.25; *P* < 0.00001; Fig. 5A). Three studies [40, 48, 52] reported results 1-month post-treatment using a fixed-effects model (*MD*: −1.40; 95% *CI*: −1.81, −0.99; *P* < 0.00001; Fig. 5B). Furthermore, five studies [40, 48, 49, 52, 53] reported 3-month posttreatment outcomes via a fixed-effects model (*MD*: −1.56; 95% *CI*: −1.92, −1.20; *P* < 0.00001; Fig. 5C). Across all three datasets, RIPostC significantly reduced the NIHSS scores compared to the control group.

#### 6-month group

**Fig. 6:**
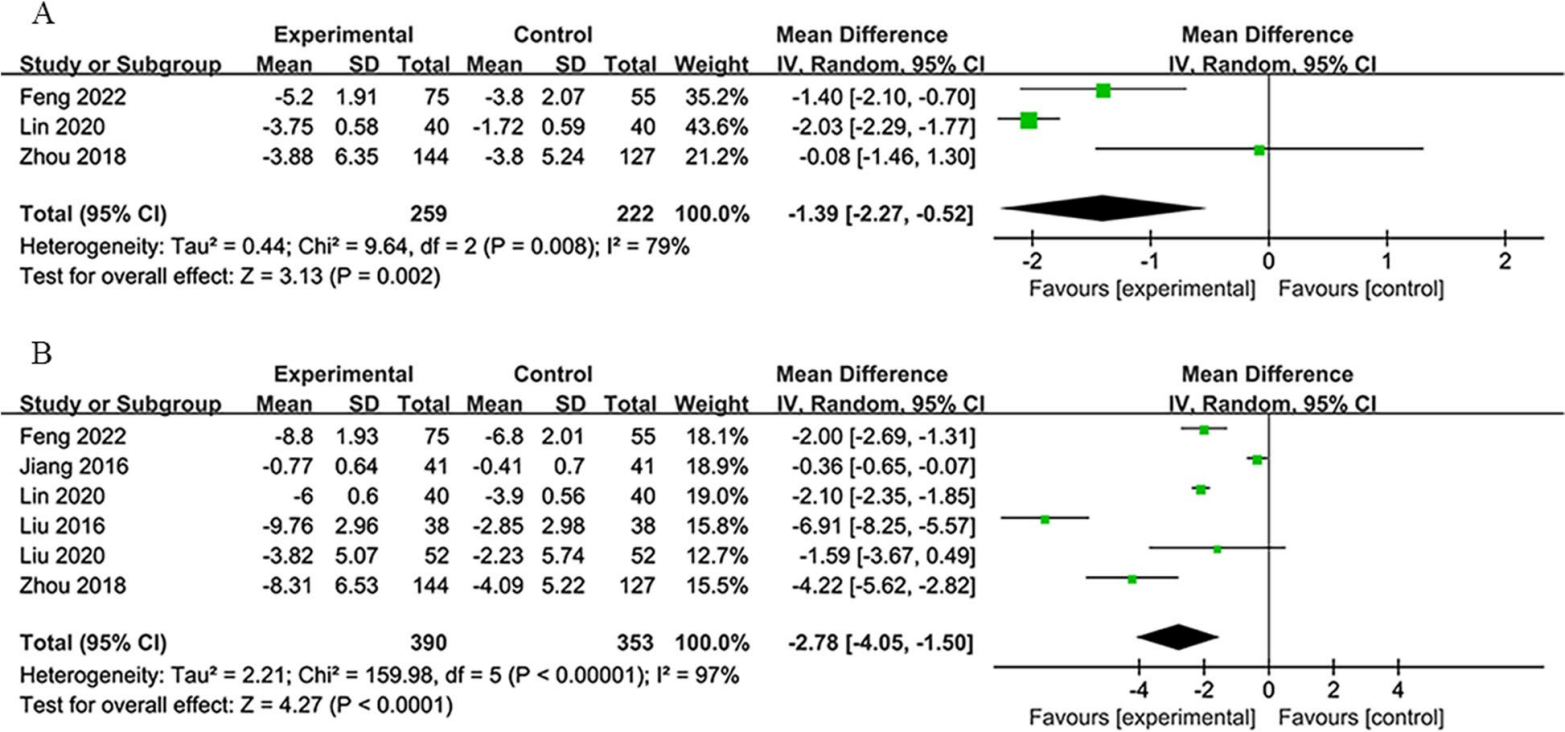
**A** Forest plot of NIHSS scores on 30th day in 6-month group. **B** Forest plot of NIHSS scores on 180th day in 6-month group

This group comprised 6 studies with 743 participants, reporting results at 1-month and 6-month post-treatment. At the 1-month mark [34, 43, 54], a meta-analysis using a random-effects model indicated that the RIPostC group outperformed the control group in reducing NIHSS scores (*MD*: −1.39; 95% *CI*: −2.27, −0.52; *P* < 0.002; Fig. 6A). In the 6th month [32–34, 39, 43, 54], NIHSS scores in the RIPostC group were significantly lower than those in the control group, as determined by a random-effects model (*MD*: −2.78; 95% *CI*: −4.05, −1.50; *P* < 0.0001; Fig. 6B).

#### Barthel index (BI)

**Fig. 7:**
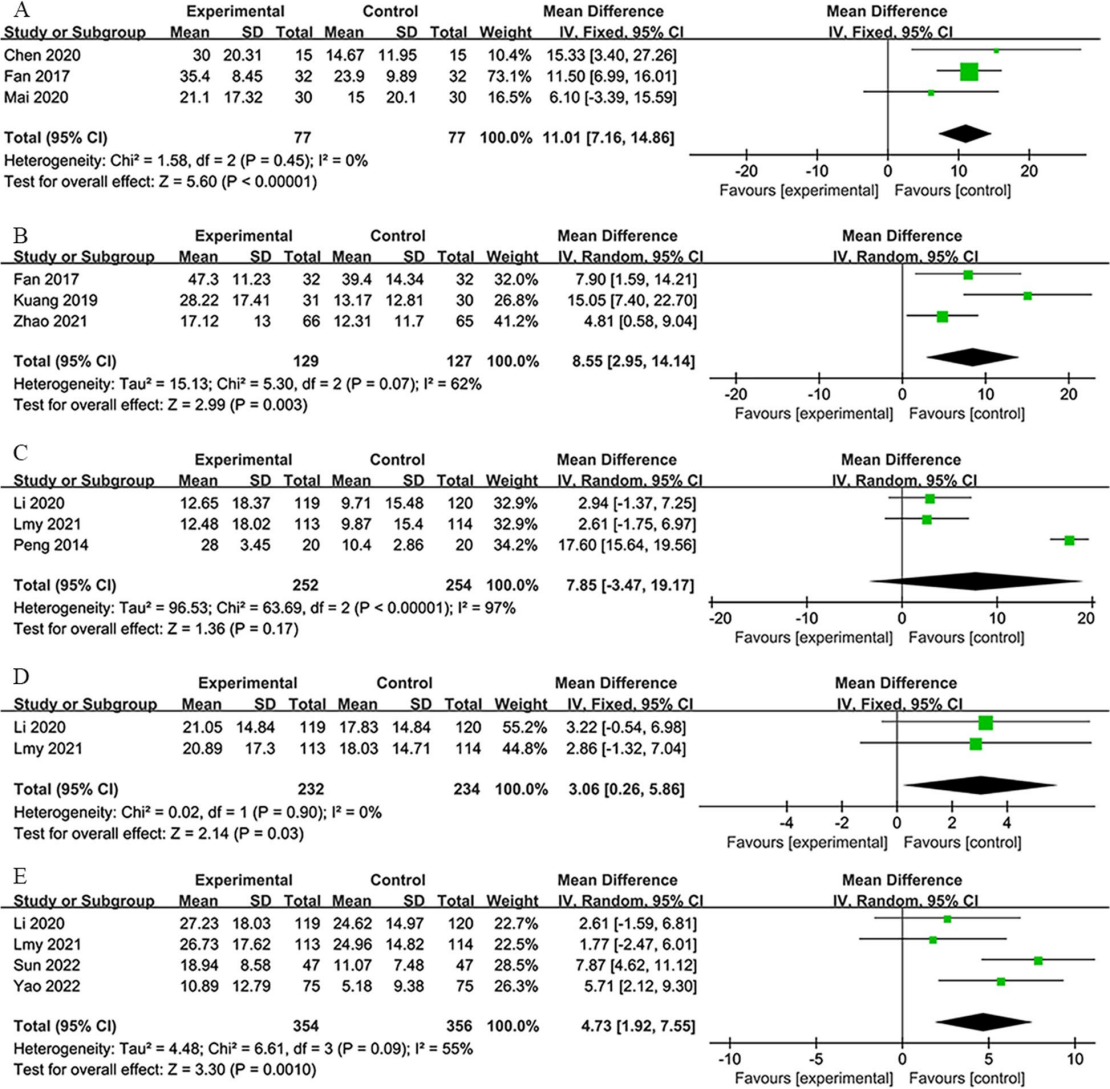
**A** Forest plot of BI on 7th day in 7-day group. **B** Forest plot of BI on 14th day in 7-day group. **C** Forest plot of BI on 14th day in 14-day group. **D** Forest plot of BI on 30th day in 14-day group. **E** Forest plot of BI on 90th day in 14-day group

For the BI, only data from the 7-day and 14-day groups were suitable for meta-analysis. The 7-day group provided outcomes on the 7th and 14th days of treatment. However, the 14-day group reported results on the 14th-, 30th-, and 90th-day post-treatment. According to Fig. 7, RIPostC significantly enhanced the patients’ BI scores compared to the control group. The BI measures the patient’s ability to engage in daily activities, with a higher score indicating more substantial independence in activities, reflecting the patient’s recovery situation.

In the 7-day group on the 7th day [29, 37, 42], the analysis showed a significant improvement (*MD*: 11.01; 95% *CI*: 7.16, 14.86; *P* < 0.00001; heterogeneity: *χ*^2^ = 1.58; *I*^2^ = 0%; *P* = 0.45; Fig. 7A).

On the 14th day [29, 35, 64], a considerable improvement was observed (*MD*: 8.55; 95% *CI*: 2.95, 14.14; *P* = 0.003; heterogeneity: *χ*^2^ =5.30; *I*^2^ = 62%; *P* = 0.07; Fig. 7B).

The 14-day group on the 14th day [4, 40, 48] experienced varied results (*MD*: 7.85; 95% *CI*: −3.47, 19.17; *P* = 0.17; heterogeneity: *χ*^2^ =63.69; *I*^2^ = 97%; *P* < 0.00001; Fig. 7C).

On the 30th day [40, 48], a slight improvement was observed (*MD*: 3.06; 95% *CI*:0.26, 5.86; *P* = 0.03; heterogeneity: *χ*^2^ = 0.02; *I*^2^ = 0%; *P* = 0.90; Fig. 7D).

By the 90th day [31, 40, 45, 53], an improvement was still evident (*MD*: 4.73; 95% *CI*:1.92, 7.55; *P* = 0.001; heterogeneity: *χ*^2^ = 6.61; *I*^2^ = 55%; *P* = 0.09; Fig. 7E).

#### Modified Rankin Scale (mRS)

For the mRS outcome metric, reports were exclusively provided by the 7-day and 14-day groups, covering treatments over 7 days and 90 days, and for the 14-day group, an additional report at 90-day post-treatment. These datasets exhibited no heterogeneity, affirming the meta-analysis results as highly dependable. Consequently, fixed-effects models were applied across the board, demonstrating that RIPostC effectively enhanced mRS scores in patients when compared to the control group. The mRS is used to evaluate the prognosis of various types of patients with stroke and to determine the efficacy of functional disability levels in rehabilitation patients. A lower score indicates a better prognosis and recovery.

**Fig. 8:**
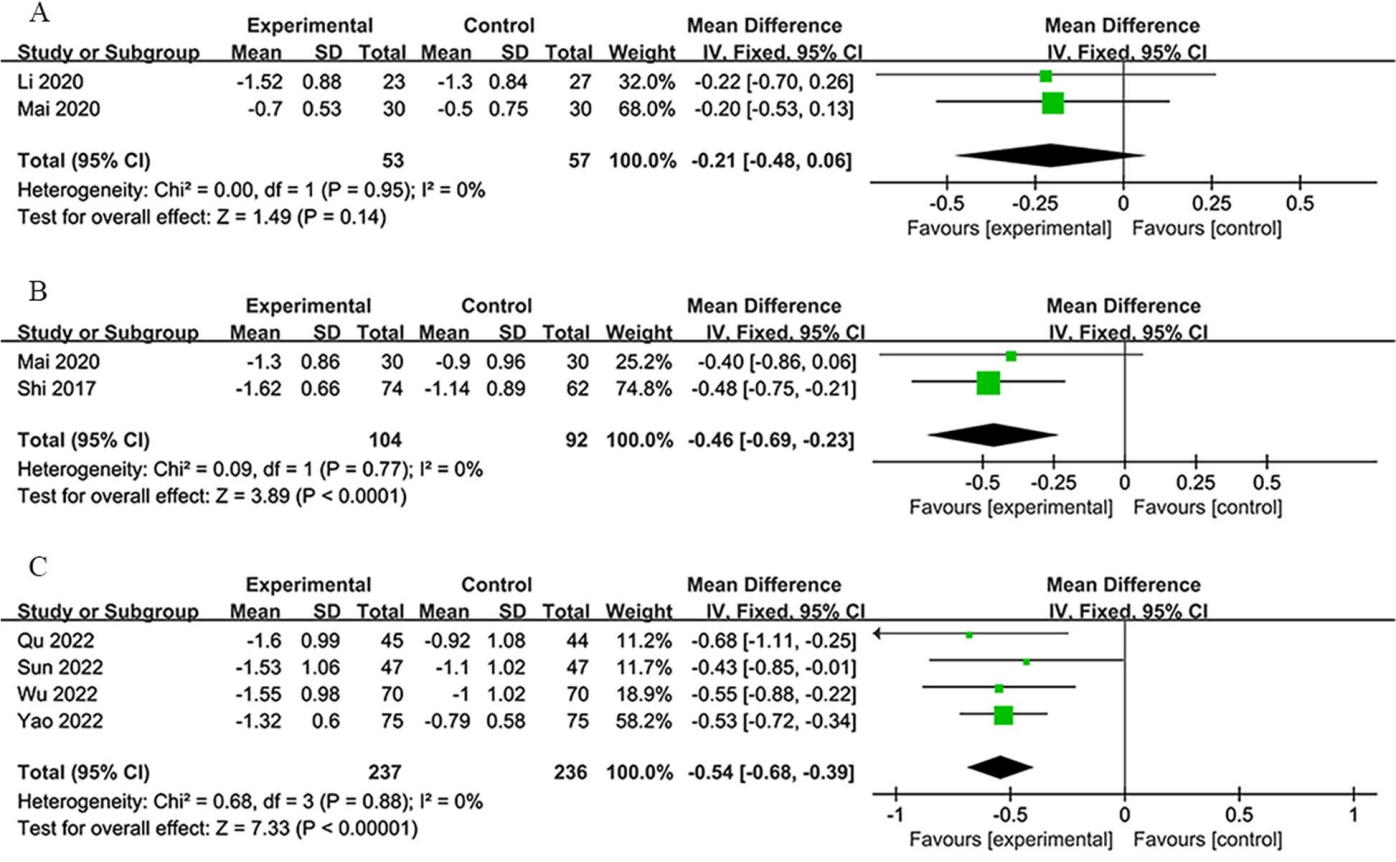
**A** Forest plot of mRS on 7th day in 7-day group. **B** Forest plot of mRS on 90th day in 7-day group. **C** Forest plot of mRS on 90th day in 14-day group

In the 7-day group at 7 days [37, 41], the analysis indicated a marginal improvement (*MD*: −0.21; 95% *CI*: −0.48, 0.06; *P* = 0.14; with zero heterogeneity: *χ*^2^ = 0.00; *I*^2^ = 0%; *P* = 0.14; Fig. 8A).

In the 7-day group at 90 days [37, 58], a significant enhancement was observed (*MD*: −0.46; 95% *CI*: −0.69, −0.23; *P* < 0.0001; with negligible heterogeneity: *χ*^2^ = 0.09; *I*^2^ = 0%; *P* = 0.77; Fig. 8B).

In the 14-day group at 90 days [31, 50, 52, 53], a considerable improvement was also recorded (*MD*: −0.54; 95% *CI*: −0.68, −0.39; *P* < 0.00001; with minimal heterogeneity: *χ*^2^ = 0.68; *I*^2^ = 0%; *P* = 0.88; Fig. 8C).

#### Interleukin-6 (IL-6) and tumor necrosis factor-α (TNF-α)

The 14-day group provided IL-6 (ng/L) levels on the 14th day of treatment, while the 6-month group reported both IL-6 and TNF-α (ng/L) levels in the first month of treatment. Despite substantial heterogeneity among these datasets, analyses using a random-effects model indicated that RIPostC significantly impacted these biomarkers favorably compared to the control group. IL-6 and TNF-α are both serum inflammatory factors, and their presence indicates inflammation. Lower levels suggest a milder inflammatory response in the patient’s body. Given the rapid inflammatory response post-stroke, the gradual decrease in inflammatory activity with treatment and time indicates stroke recovery.

**Fig. 9:**
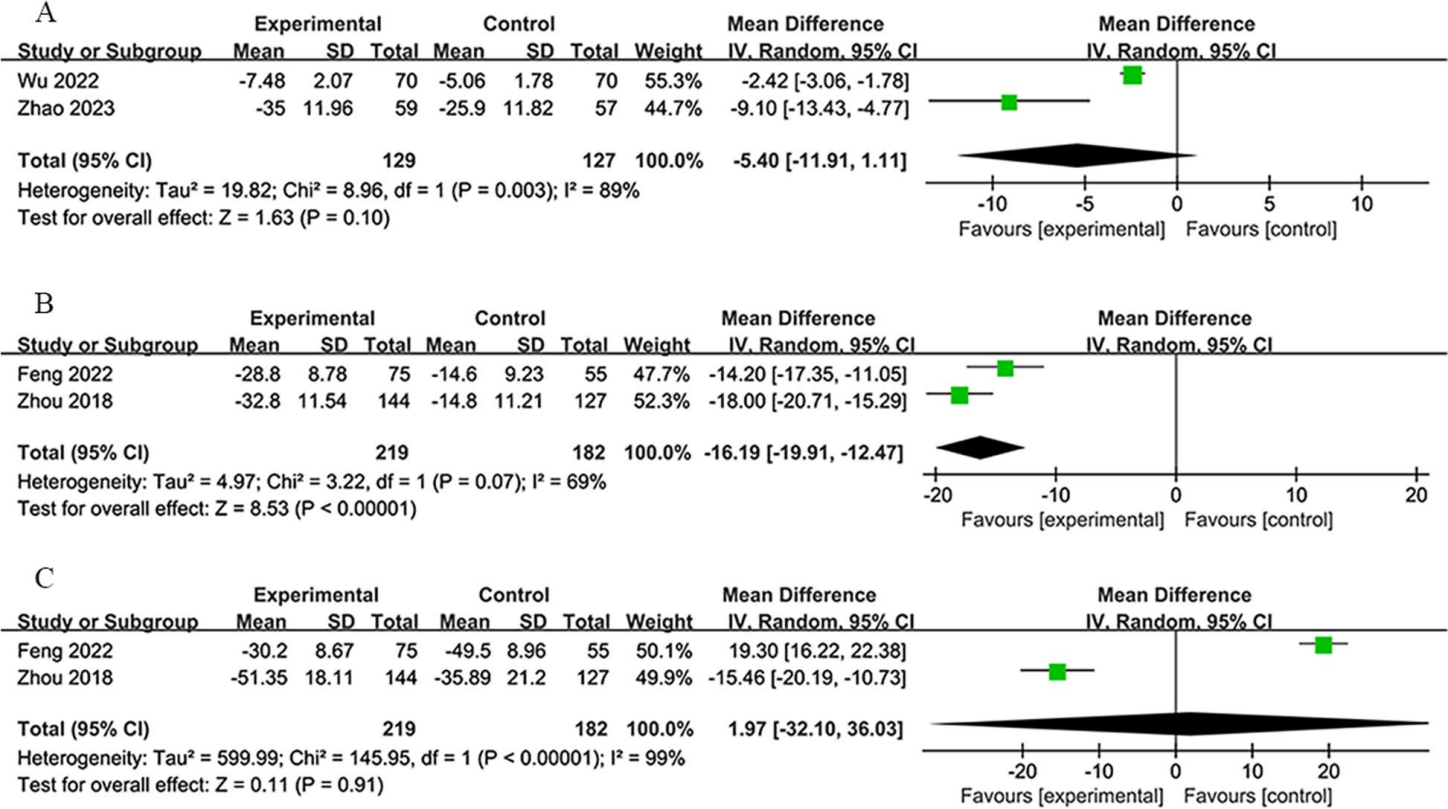
**A** Forest plot of IL-6 on 14th day in 14-day group. **B** Forest plot of IL-6 on 30th day in 6-month group. **C** Forest plot of TNF-α on 30th day in 6-month group

At the 14-day mark for the 14-day group [50, 56], IL-6 levels considerably decreased (*MD*: −5.40; 95% *CI*: −11.91, 1.11; *P* = 0.10; heterogeneity: *χ*^2^ = 8.96; *I*^2^ = 89%; *P* = 0.003; Fig. 9A).

At the 30-day mark for the 6-month group [34, 54], IL-6 levels significantly reduced (*MD*: −16.19; 95% *CI*: −19.91, −12.47; *P* < 0.00001; heterogeneity: *χ*^2^ = 3.22; *I*^2^ = 69%; *P* = 0.07; Fig. 9B).

For TNF-α at the 30-day mark [34, 54], significant changes were not observed (*MD*: 1.97; 95% *CI*: −32.10, 36.03; *P* = 0.91; heterogeneity: *χ*^2^ = 145.95; *I*^2^ = 99%; *P* < 0.00001; Fig. 9C).

#### C-reactive protein (CRP), fibrinogen (FIB), and D-dimer (D-D) levels

**Fig. 10:**
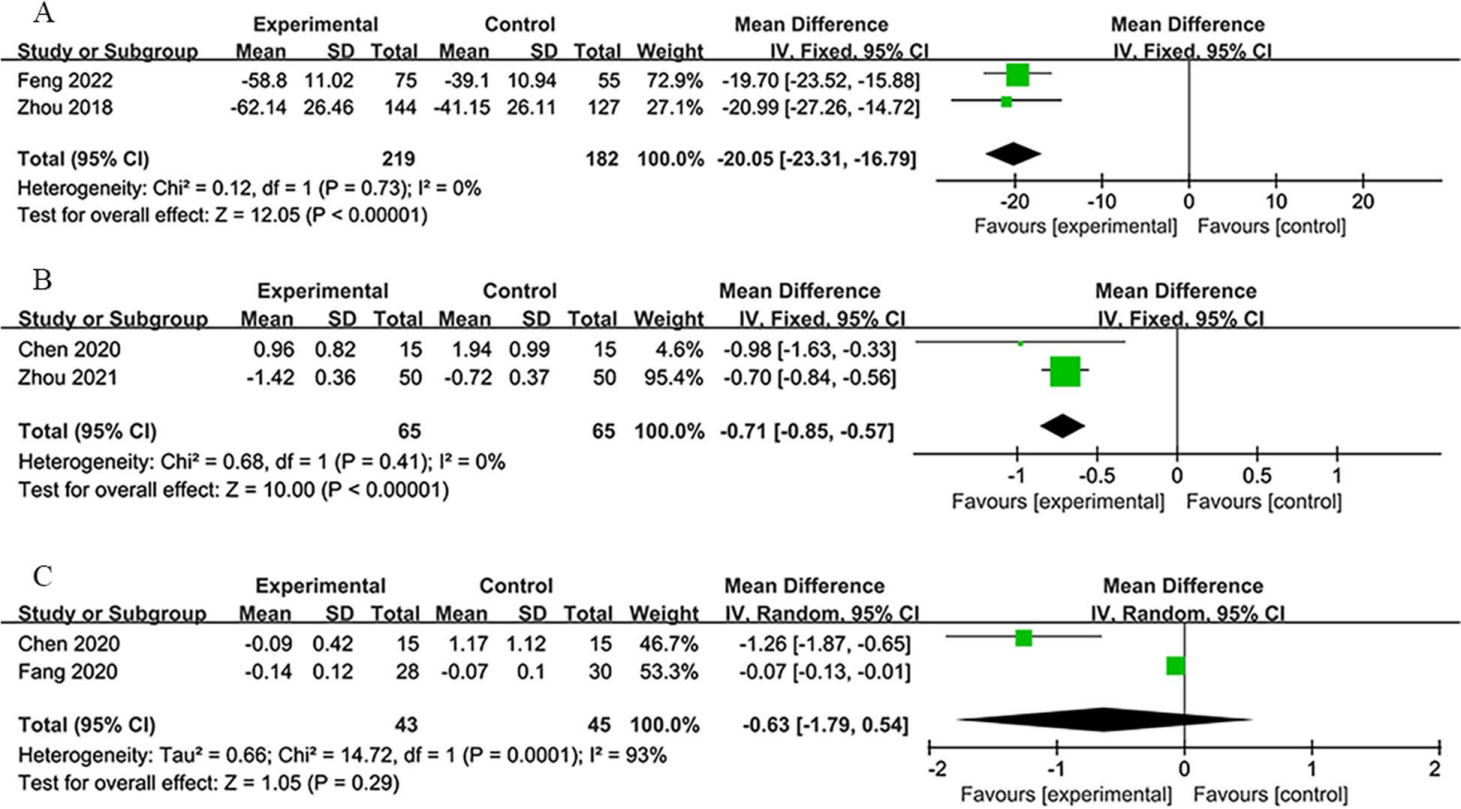
**A** Forest plot of CRP on 30th day in 6-month group. **B** Forest plot of FIB on 7th day in 7-day group. **B** Forest plot of D-D on 7th day in 7-day group

CRP (pg/L) levels reported by the 6-month group at 1 month showed significant improvement [34, 54] (*MD*: −20.05; 95% *CI*: −23.31, −16.79; *P* < 0.00001; heterogeneity: *χ*^2^ = 0.12; *I*^2^ = 0%; *P* = 0.73; Fig. 12A). FIB (g/L) levels, disclosed by the 7-day group. improved significantly [42, 47] (*MD*: −0.71; 95% *CI*: −0.85, −0.57; *P* < 0.00001; heterogeneity: *χ*^2^ = 0.68; *I*^2^ = 0%; *P* = 0.41). D-D (mg/L) levels reported by the 7-day group indicated an insignificant decrease [36, 42] (*MD*: −0.63; 95% *CI*: −1.79, 0.54; *P* = 0.29; heterogeneity: *χ*^2^ = 14.72; *I*^2^ = 93%; *P* = 0.0001). Figure 10 illustrates these findings.

CRP is related to the inflammatory response, and the decrease in this indicator reflects the disappearance of inflammation in the patient’s body. The increase in FIB level represents the hypercoagulable state of blood, which is a critical factor in the occurrence of cerebral infarction and hinders the recovery of brain tissue after cerebral infarction. Its decrease represents the gradual withdrawal of the patient’s blood from the hypercoagulable state, which benefits the patient’s recovery. D-D mainly reflects the fibrinolytic function. An increase in D-D levels in plasma indicates the presence of secondary fibrinolysis, with lower levels indicating a better prognosis. These results suggest that RIPostC can modify these three indicators beneficially, although heterogeneity across studies warrants careful interpretation of these results.

#### Brain-derived neurotrophic factor (BDNF)

**Fig. 11:**
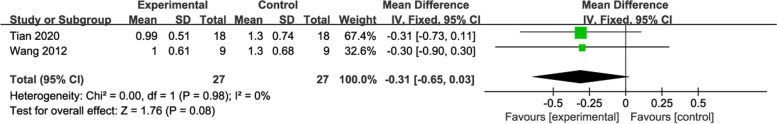
Forest plot of BDNF on 14th day in 14-day group

The 14-day group provided outcomes for BDNF at 14-day post-treatment [60, 61]. The meta-analysis improved significantly in BDNF (µg/L) compared to the control group. Figure 11 illustrates the following finding. A marginal reduction was noted in the BDNF levels (*MD*: −0.31; 95% *CI*: −0.65, 0.03; *P* = 0.08; with no heterogeneity: *χ*^2^ = 0.00; *I*^2^ =0%; *P* = 0.98; Fig.e 11). The minimal change and close-to-significance *P*-value indicate a modest effect of RIPostC on increasing BDNF levels, albeit without statistical significance. BDNF regulates growth and development, and induces differentiation, while modulating the synaptic connections of embryonic neurons. It simultaneously participates in activity-dependent neuronal plasticity, including regeneration, repair, and protection after injury, especially for cognitive function-related areas such as the frontal lobe and hippocampus. Therefore, higher levels of BDNF are expected to be more beneficial for patients.

### Risk-of-*bias* assessment in the individual study

**Fig. 12:**
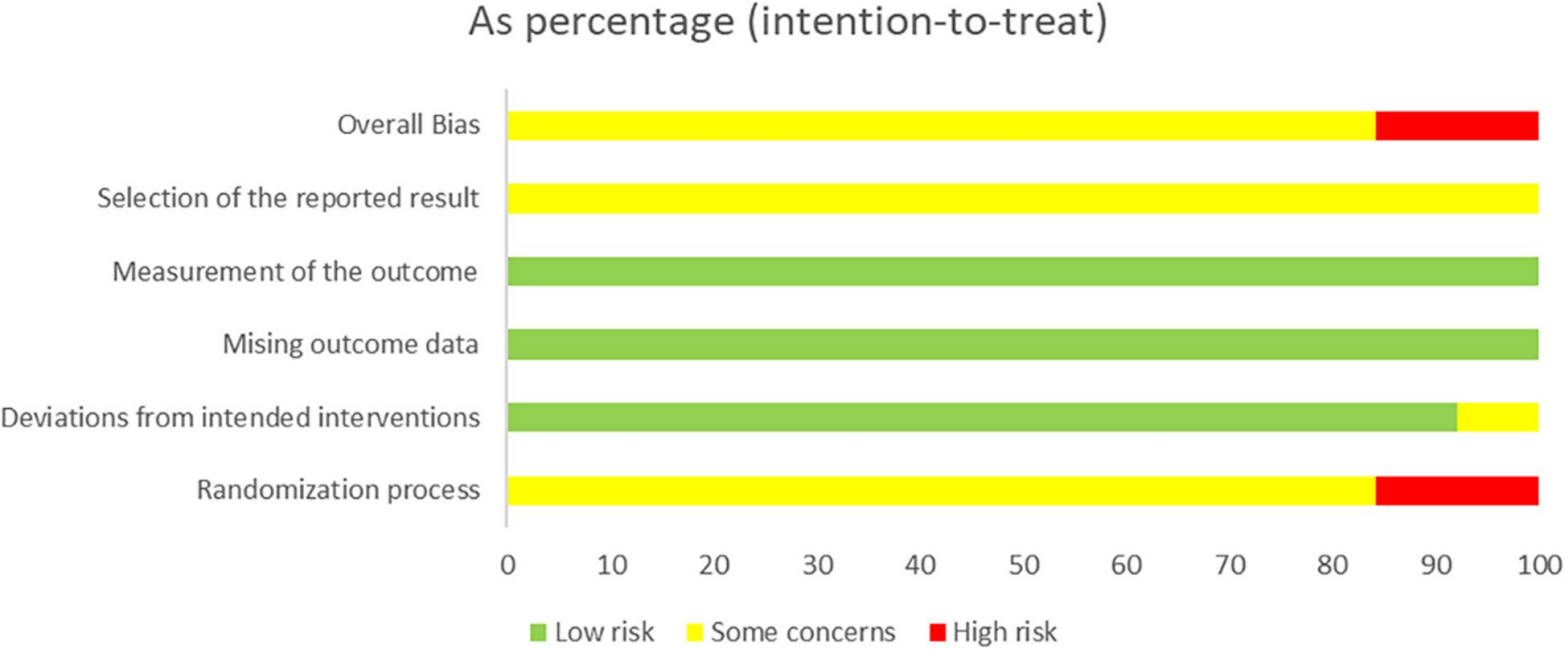
Risk-of-bias graph

**Fig. 13:**
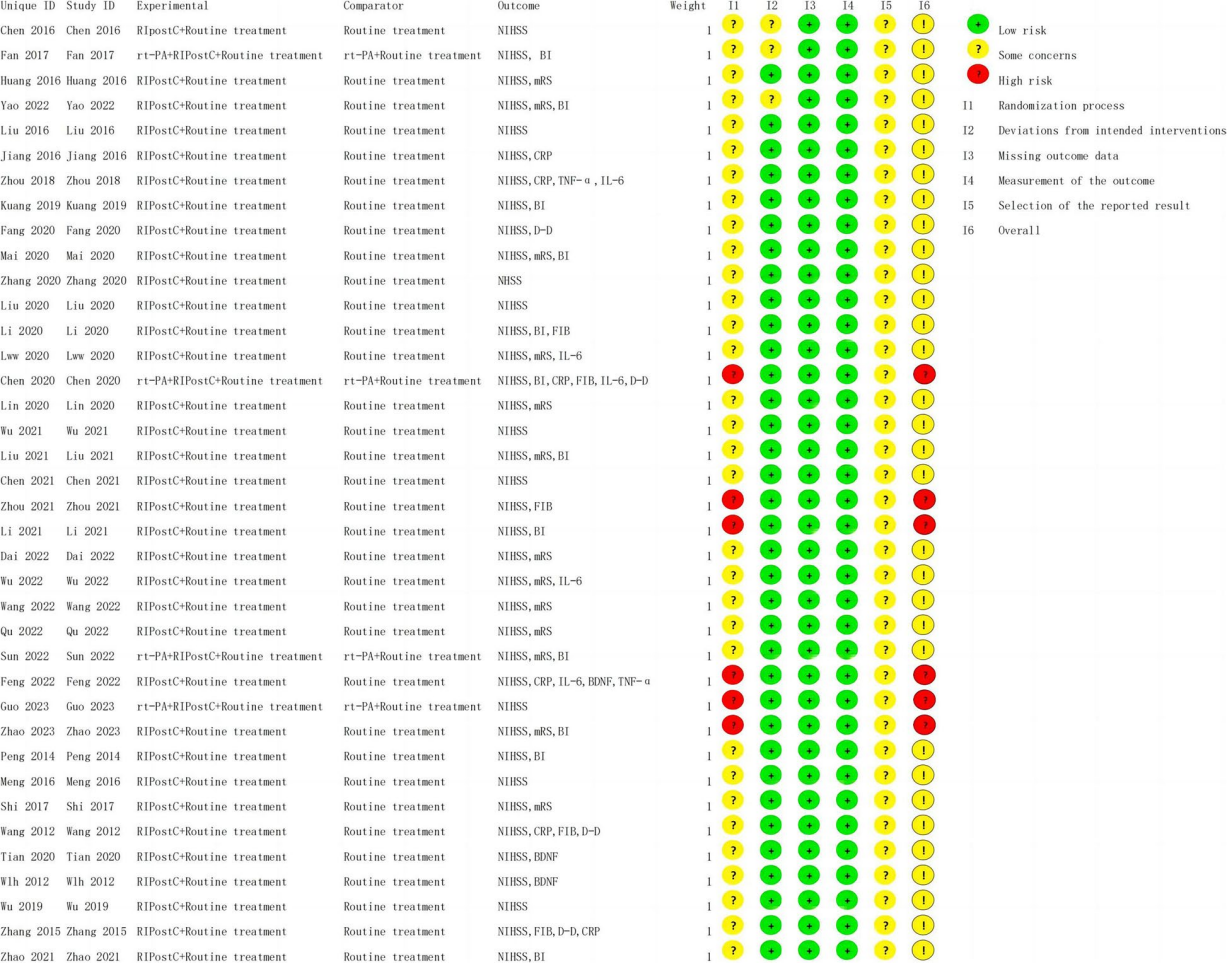
Risk-of-bias summary graph

Cochrane Evaluation Tool 2 (ROB2) was used to evaluate the risk of bias in all included studies, covering five areas: bias risk caused by the randomization process, bias risk caused by deviation from expected intervention measures, missing outcome data, the risk of bias in outcome measurement, and the risk of bias in reporting outcome selection [65]. These areas are divided into “low risk,” “high risk,” or “some concerns”; they are independently completed by two researchers (M. Y. and J. W.), with a third researcher (J. L.) discussing and resolving potential disagreements. The quality assessment of the studies is shown in Figs. 12 and 13. The Additional files 6 and 7 lists specific details (see Additional files 6 and 7).

### Sensitivity analysis

Data analyses revealed heterogeneity across nearly all results, leading to using a random-effects model for the meta-analysis. Identifying the precise contributors to this heterogeneity proved challenging even after sensitivity testing was performed. The observed heterogeneity is hypothesized to significantly correlate with factors such as the level of care provided by local hospitals, patient demographics, diversity in treatment approaches, and post-disease management. Despite this heterogeneity, the data suggest that RIPostC is an effective adjuvant therapy. Consequently, this analysis does not delve further into the issue of heterogeneity.

### Publication *bias*

The potential for publication bias was investigated in the 14-day group, which focused solely on the NIHSS score as an outcome measure and included over 10 original studies. The analysis suggested the likelihood of publication bias, which may stem from the small sample sizes of the numerous included studies and the potential for selective reporting by the researchers. The overall quality of the included studies was assessed as low, complicating the identification of the source of this publication bias. The funnel plot detailing this analysis is available in the Additional file 8 (see Additional file 8).

### GRADE evidence quality

The evidence quality for the outcomes assessed in the systematic review and meta-analysis was evaluated using the GRADE approach. The mRS was deemed to be of “moderate” quality. In contrast, the NIHSS, BI, and CRP were determined to be of low quality. Furthermore, IL-6, TNF-α, FIB, D-D, and BDNF received “very low” quality ratings. The Additional file 9 details an in-depth grading analysis (see Additional file 9).

### Adverse reactions

Adverse reactions were reported in only five studies [33, 36, 47, 48, 64]. Most studies did not report any adverse reactions or were devoid of them. In Jiang et al.’s research [33], two patients reported minor swelling and pain in the limb used for pressure training, which subsided completely after a 30-min rest period. Fang et al.’s [36] study noted a single case of limb numbness in the experimental group, which resolved during treatment. Zhou et al. [47] reported that two patients who gradually adapted initially experienced pain in the upper limbs during training. According to Zhao et al.’s investigation [64], 32 patients experienced pain and numbness in the compressed limb during the RIPostC operation, 4 of whom exhibited subcutaneous bleeding; these symptoms were relieved entirely within 5-min postoperation, and the bruising resolved within a day. Li et al. [48] reported that five patients had bleeding spots and petechiae at the cuff site following treatment, which naturally resolved within 1 to 2 days; three patients developed numbness and swelling in the upper arm, which reduced or vanished upon cuff adjustment or pressure reduction, becoming tolerable after 2 to 3 days of acclimatization.

## Discussion

In this meta-analysis, randomized controlled trials of RIPostC were systematically reviewed to assess its efficacy in enhancing the salvage and recovery of patients suffering from AIS. Compared to control groups, RIPostC demonstrated superior outcomes across most measured indicators, showing significant advantages. This meta-analysis included 38 randomized controlled trials involving 4334 patients. The intervention groups underwent RIPostC, while the control groups were treated with standard therapeutic interventions such as thrombolysis, blood pressure management, anticoagulation, antiplatelet therapy, antioxidant treatments, and fluid replenishment. The statistical outcomes revealed that RIPostC significantly reduced the NIHSS scores in patients with acute cerebral infarction, independent of the duration of the intervention or the specific protocols of RIPostC applied. Regarding the BI scores, though only the 7-day and 14-day intervention data were eligible for this meta-analysis, it was observed that RIPostC contributed to significant improvements in BI scores over both short-term (7-day and 14-day) and long-term (90-day) periods. Similarly, the mRS analysis, restricted to the 7-day and 14-day data, indicated that RIPostC effectively enhanced mRS scores in short-term (7-day) and long-term (90-day) assessments, suggesting an improved prognosis for patients receiving RIPostC treatment. In acute inflammation, analyses incorporated IL-6 data from the 14-day and 6-month groups. The findings indicated a significant decrease in IL-6 levels by the 30th day compared to the 14th day, indicating that extended treatment duration with RIPostC enhances anti-inflammatory recovery in patients. TNF-α levels were explicitly reported by the 6-month group, demonstrating RIPostC’s effectiveness. A comparison with shorter-duration data, similar to the IL-6 analysis, would have provided a clearer picture of RIPostC’s impact on reducing TNF-α levels. CRP serves as a marker for chronic inflammation related to atherosclerosis. CRP levels, reported by the 6-month group at 1 month, showed a reduction following RIPostC treatment. Future research should include assessments at varied intervals to comprehensively assess RIPostC’s effect on atherosclerosis-related chronic inflammation. FIB and D-D are indicators of the hypercoagulable state in patients with AIS. Both indicators, reported by the 7-day group on the 7th day, showed reductions post-RIPostC intervention, indicating amelioration of the hypercoagulable state. The limited measurement time points highlight the need for future studies to incorporate multiple assessment moments to depict changes fully. The elevation of BDNF concentration in the cortex could enhance synaptogenesis and dendritic spine development, thereby facilitating cortical functional remodeling in survivors of stroke. Despite only the 14-day group reporting on BDNF levels at 14 days, RIPostC was observed to increase this parameter, suggesting facilitation in the neuronal repair process in patients. Regarding the safety of RIPostC, adverse effects were reported only in five studies, with all incidents being minor and not impacting the course of treatment. This observation suggests that RIPostC is safe, with no cases of recurrent cerebral infarction or death reported among participants across the studies.

RIPostC has been more frequently applied in cardiac ischemia [66–70]; however, its use in treating cerebral ischemic conditions has increased recently. The protective action of RIPostC in patients with ischemia is multifaceted, involving anti-inflammatory and antioxidant effects, inhibition of apoptosis, regulation of protein expression, and modulation of protease activity [71]. These effects activate the body’s innate ischemic tolerance through short cycles of nonfatal, reversible ischemia and reperfusion, mediated by both humoral and immune-inflammatory regulatory mechanisms, conferring protection. Although the serum factors measured in the included studies do not fully cover all aspects of RIPostC’s protective mechanisms, they illustrate its potential benefits. FIB is a coagulation factor in plasma and an inflammatory marker, contributing to atherosclerosis by accumulating in the vessel wall, which can lead to endothelial cell migration, denaturation, and thrombosis due to its role in smooth muscle cell proliferation, hypertrophy, and enhancement of platelet aggregation and blood viscosity. Elevated levels of FIB are closely associated with the onset of acute cerebral infarction. D-D indicates fibrinolytic activity, with its levels increasing in response to active thrombus formation and fibrinolysis within the body’s blood vessels. The presence of covalent cross-links between the Y-chains underpins the structure of D-D, making it a sensitive and specific marker for the early diagnosis of cerebral infarction. Moreover, D-D levels help monitor the effectiveness of thrombolytic therapy and are directly proportional to the severity of cerebral infarction. Patients exhibiting elevated D-D levels in their plasma are at an increased risk of experiencing subsequent cerebral infarctions [72–75]. Research has documented that an inflammatory cascade is triggered following ischemia-reperfusion injury in the brain. CRP is an inflammatory marker capable of independently predicting future vascular incidents. Elevated CRP levels may serve as an indirect indicator of endothelial cell impairment, activation of inflammatory cytokines, vascular damage, and the predominant thrombotic conditions, all of which are intricately linked to the development and progression of atherosclerosis [76–80]. BDNF is a neurotrophic factor critical in maintaining neuronal survival, growth, differentiation, and repair post-injury. It enhances the proliferation of neural stem cells within the brain, fostering the developmental differentiation and growth necessary for neuronal regeneration. BDNF levels are significantly crucial for the recovery process in patients with ischemic stroke [81–83]. Beyond the direct impact of cerebral ischemia due to the depletion of intermediate metabolites and oxygen, the release of various amino acids from the cytosol substantially contributes to postischemic brain damage. The metrics analyzed in this study, including the NIHSS, BI, mRS, IL-6, TNF-α, CRP, FIB, D-D, and BDNF, demonstrate either improvement or reduction due to RIPostC over extended intervention durations. These observations indicate that the benefits of RIPostC become more pronounced with extended intervention periods, highlighting its potential as a treatment for AIS.

The considerable heterogeneity observed across groups for several indicators could stem from substantial differences in either the baseline or endpoint values of these indicators in certain studies, differences in the standard treatments administered, or variations in the duration, number of cycles, timing of ischemia, and reperfusion periods used in RIPostC across studies. Despite these sources of heterogeneity, the findings from the random-effects model meta-analysis are deemed significant.

### Comparison with previous studies

In exploring English-language databases, only four meta-analyses and systematic reviews relevant to this field have been identified, with two focusing on animal studies and the other on clinical trials. The reviews of animal studies [84, 85], though encompassing a wide array of primary research and conducted across various species, provided a comprehensive and detailed assessment of the outcomes. These analyses present robust evidence supporting the preclinical efficacy of RIPostC. However, these two animal studies could interest researchers intending to conduct animal-based investigations into RIPostC. The remaining two reviews, focusing on clinical trials, one published in 2022 [86] and the other in 2018 [87], offer crucial insights and serve as significant resources for clinical practitioners during those periods. Both reviews confirmed the therapeutic significance and safety of RIPostC in the treatment of patients with ischemic stroke. However, they were limited by the relatively small number of primary studies they encompassed, and the scope of outcome measures considered was not exhaustive. Furthermore, subgroup analyses were not conducted. While recognizing the significance of these two reviews in evidence-based medicine and their relevance for clinical practitioners, this study incorporates more primary research, a broader range of outcome measures, and subgroup discussions, enhancing the robustness of the analytical results.

### Strengths of this study

This review compiles all existing research on RIPostC for AIS treatment in China, categorizing it into detailed segments based on the duration of RIPostC application. It examines up to 10 outcome indicators to elucidate the effects of RIPostC as comprehensively as possible. While medical professionals have recommended RIPostC for cardiac conditions for many years, evidence has highlighted its significant efficacy in stroke treatment. In an era where alternative stroke treatments are actively sought, RIPostC emerges as an effective, safe, and novel adjuvant therapy. This review contributes new evidence-based medical insights for the clinical application of RIPostC, supporting its application beyond traditional heart disease treatments.

### Limitations

This study may exhibit a language bias due to the exclusive inclusion of Chinese-language studies. The methodological quality of the included studies was generally low, with no descriptions of allocation concealment or blinding provided, raising concerns about potential researcher bias. Variations in the frequency, pressure of RIPostC application, intervention durations, and inconsistencies in the reported underlying treatments likely contributed to the high heterogeneity across studies. With a total sample size of 4324 participants across single-center trials, the study’s results could be influenced by the subjective perspectives of the researchers, impacting the robustness of the conclusions. The potential effect of the patients’ preexisting conditions on the outcome indicators further limits the extrapolation of the study’s findings. Adverse reactions were reported in only five studies, indicating that a more comprehensive safety assessment across more extensive and diverse clinical studies is warranted. The publication bias, possibly resulting from small study sizes, lack of multicenter trials, and selective reporting by researchers, cannot be ruled out. The overall low quality of the included studies complicates identifying specific causes for this issue.

## Conclusions

In summary, the available evidence suggests that RIpostC beneficially impacts the treatment and recovery of patients with acute cerebral infarction compared to standard controls. However, the inherent limitations in the design quality of the included trials may influence the outcomes of our analysis, introducing a degree of bias. Consequently, future research should focus on elucidating the mechanistic underpinnings of RIpostC’s effects on AIS. Furthermore, there is a need for further randomized controlled trials employing rigorous methodologies to substantiate our findings.

### Supplementary Information

Additional file 1. Specific search strategies.

Additional file 2. Data Extraction Checklist.

Additional file 3. Raw data.

Additional file 4. Calculated data Table 1.

Additional file 5. Calculated data Table 2.

Additional file 6. ROB 2_JWR2.

Additional file 7. ROB 2_JWR2.

Additional file 8. Publication bias plot.

Additional file 9. GRADE evidence quality.

Additional file 10. PRISMA checklist.

## Data Availability

The original contributions presented in the study are included in the article, further inquiries can be directed to the corresponding author.
