# Supplementary material for: Impact of remote ischemic postconditioning on acute ischemic stroke in China: a systematic review and meta-analysis of randomized controlled trials
Source: Syst Rev. 2024 May 30;13:141. doi: 10.1186/s13643-024-02568-3 (PMC11138007; doi:10.1186/s13643-024-02568-3)
Supplement: Supplementary file 1 — Additional file 1. Specific search strategies. [file 13643_2024_2568_MOESM1_ESM.docx]

**CNKI/高级检索; CNKI / Advanced Search**

主题:(脑梗死 OR 急性脑梗死 OR 中风 OR 缺血性中风OR 缺血性脑卒中) AND (远隔缺血后适应 OR 远隔缺血适应 OR 远程缺血后适应 OR 远程缺血适应 OR 远隔缺血预适应 OR 远程缺血预适应) AND (随机对照试验) (52)

Subject: (cerebral infarction OR acute ischemic stroke OR stroke OR Ischemic stroke) AND (remote ischemic postconditioning OR remote ischemic conditioning OR remote ischemic preconditioning) AND (randomized controlled trial) (52)

**万方/高级检索; Wanfang database/ Advanced Search**

主题:(脑梗死 OR 急性脑梗死 OR 中风 OR 缺血性中风OR 缺血性脑卒中) AND (远隔缺血后适应 OR 远隔缺血适应 OR 远程缺血后适应 OR 远程缺血适应 OR 远隔缺血预适应 OR 远程缺血预适应) AND (随机对照试验) (84)

Subject: (cerebral infarction OR acute ischemic stroke OR stroke OR Ischemic stroke) AND (remote ischemic postconditioning OR remote ischemic conditioning OR remote ischemic preconditioning) AND (randomized controlled trial) (84)

**VIP/高级检索; VIP database/ Advanced Search**

题名或关键词: (脑梗死 OR 急性脑梗死 OR 中风 OR 缺血性中风 OR 缺血性脑卒中) AND (远隔缺血后适应 OR 远隔缺血适应 OR 远程缺血后适应 OR 远程缺血适应 OR 远隔缺血预适应 OR 远程缺血预适应) AND (随机对照试验) (27)

Title or keyword: (cerebral infarction OR acute ischemic stroke OR stroke OR Ischemic stroke) AND (remote ischemic postconditioning OR remote ischemic conditioning OR remote ischemic preconditioning) AND (randomized controlled trial) (27)

**CBM/高级检索; CBM / Advanced Search**

全部字段:(脑梗死 OR 急性脑梗死 OR 中风 OR 缺血性中风OR 缺血性脑卒中) AND (远隔缺血后适应 OR 远隔缺血适应 OR 远程缺血后适应 OR 远程缺血适应 OR 远隔缺血预适应 OR 远程缺血预适应) AND (随机对照试验) (17)

All fields: (cerebral infarction OR acute ischemic stroke OR stroke OR Ischemic stroke) AND (remote ischemic postconditioning OR remote ischemic conditioning OR remote ischemic preconditioning) AND (randomized controlled trial) (17)

**Pubmed**/**Advanced Search**

#1"acute ischemic stroke"[Title/Abstract] OR "ischemic strokes"[Title/Abstract] OR "stroke ischemic"[Title/Abstract] OR "ischaemic stroke"[Title/Abstract] OR "ischaemic strokes"[Title/Abstract] OR "stroke ischaemic"[Title/Abstract] OR "cerebral infarction"[Title/Abstract] OR "cerebral infarct"[Title/Abstract] 55885

#2"remote ischemic postconditioning"[Title/Abstract] OR "remote ischemic conditioning"[Title/Abstract] OR "RIPostC"[Title/Abstract] 634

#3("acute ischemic stroke"[Title/Abstract] OR "ischemic strokes"[Title/Abstract] OR "stroke ischemic"[Title/Abstract] OR "ischaemic stroke"[Title/Abstract] OR "ischaemic strokes"[Title/Abstract] OR "stroke ischaemic"[Title/Abstract] OR "cerebral infarction"[Title/Abstract] OR "cerebral infarct"[Title/Abstract]) AND ("remote ischemic postconditioning"[Title/Abstract] OR "remote ischemic conditioning"[Title/Abstract] OR "RIPostC"[Title/Abstract]) 80

**Embase**/**Advanced Search**

#4. #1 AND #2 AND #3 82

#3. 'randomized controlled trial' 1,105,498

#2. 'remote ischemic postconditioning' OR 'remote 2,892

ischemic conditioning' OR 'remote ischemic

preconditioning'

#1. 'acute ischemic stroke'/exp OR 'acute ischemic 175,884

stroke' OR 'ischemic strokes' OR 'stroke

ischemic' OR 'ischaemic stroke'/exp OR 'ischaemic

stroke' OR 'ischaemic strokes' OR 'stroke

ischaemic' OR 'cerebral infarction'/exp OR

'cerebral infarction' OR 'cerebral infarct'/exp

OR 'cerebral infarct'

**Web of Science**/**Advanced Search**

1: TS=(acute ischemic stroke OR ischemic strokes OR stroke ischemic OR ischaemic stroke OR ischaemic strokes OR stroke ischaemic OR cerebral infarction OR cerebral infarct) Results: 146810

2: TS=(remote ischemic postconditioning OR remote ischemic conditioning OR remote ischemic preconditioning) Results: 2231

3: #1 AND #2 Results: 426

4: TS=(randomized controlled trial) Results: 464524

5: #3 AND #4 Results: 93
