# Supplementary figures and images for: Impact of remote ischemic postconditioning on acute ischemic stroke in China: a systematic review and meta-analysis of randomized controlled trials
Source: Syst Rev. 2024 May 30;13:141. doi: 10.1186/s13643-024-02568-3 (PMC11138007; doi:10.1186/s13643-024-02568-3)

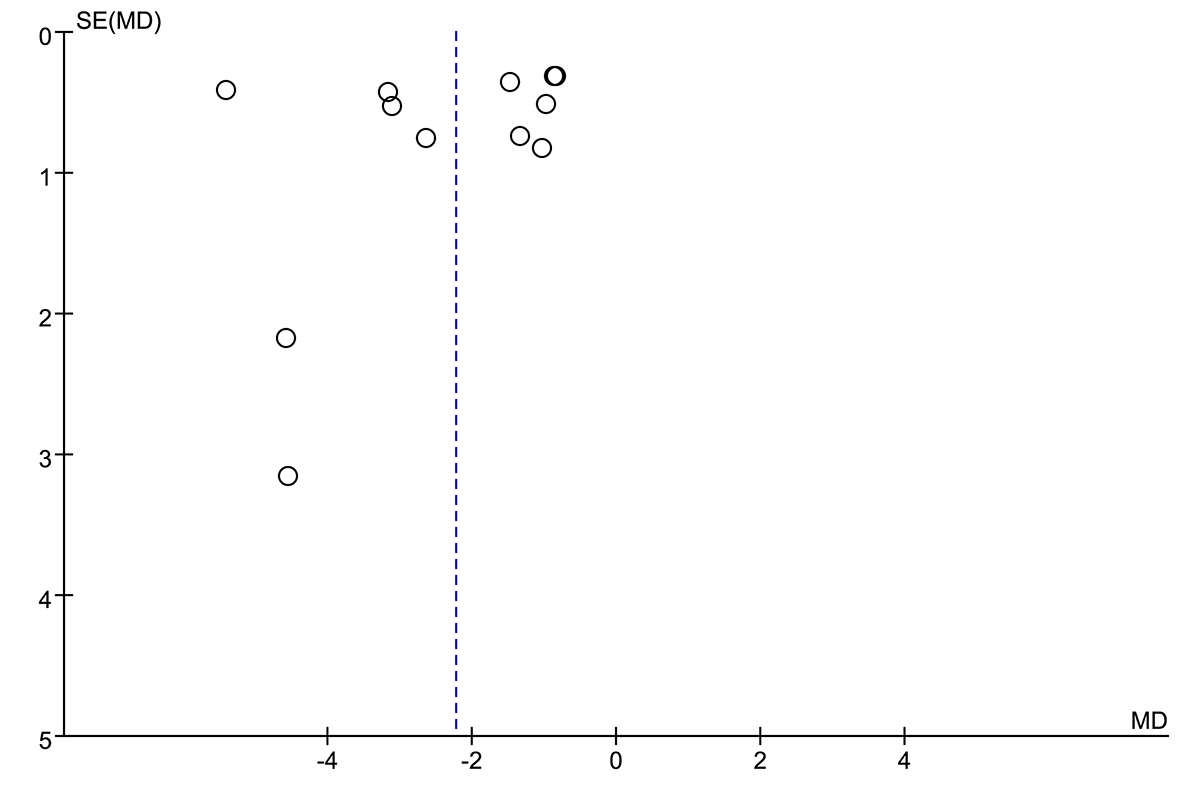

Supplement: Supplementary file 8 — Additional file 8. Publication bias plot. [file 13643_2024_2568_MOESM8_ESM.tif]
