# Supplementary material for: Impact of remote ischemic postconditioning on acute ischemic stroke in China: a systematic review and meta-analysis of randomized controlled trials
Source: Syst Rev. 2024 May 30;13:141. doi: 10.1186/s13643-024-02568-3 (PMC11138007; doi:10.1186/s13643-024-02568-3)
Supplement: Supplementary file 9 — Additional file 9. GRADE evidence quality. [file 13643_2024_2568_MOESM9_ESM.docx]

| Outcomes | Number of studies | Sample size | Evidence quality evaluation | | | | | Level of Evidence |
| --- | --- | --- | --- | --- | --- | --- | --- | --- |
|  |  | Total (RIPostC/Control) | Risk of bias | Inconsistency | Indirection | Inaccuracy | Other |  |
| NIHSS | 38 | 4324 (2158/2166) | Downgraded 1 level 1) | Not downgraded | Not downgraded | Not downgraded | Downgraded 1 level 4) | ++ |
| BI | 10 | 1136 (568/568) | Downgraded 1 level 1) | Not downgraded | Not downgraded | Not downgraded | Downgraded 1 level 4) | ++ |
| mRS | 7 | 645 (290/355) | Downgraded 1 level 1) | Not downgraded | Not downgraded | Not downgraded | Not downgraded | +++ |
| IL-6 | 4 | 657 (348/309) | Downgraded 1 level 1) | Downgraded 1 level 2) | Not downgraded | Not downgraded | Downgraded 1 level 4) | + |
| TNF-α | 2 | 401 (219/182) | Downgraded 1 level 1) | Downgraded 1 level 2) | Not downgraded | Not downgraded | Downgraded 1 level 4) | + |
| CRP | 2 | 401 (219/182) | Downgraded 1 level 1) | Downgraded 1 level 2) | Not downgraded | Not downgraded | Not downgraded | ++ |
| FIB | 2 | 130 (65/65) | Downgraded 1 level 1) | Downgraded 1 level 2) | Not downgraded | Downgraded 1 level 3) | Not downgraded | + |
| D-D | 2 | 88 (43/45) | Downgraded 1 level 1) | Downgraded 1 level 2) | Not downgraded | Downgraded 1 level 3) | Downgraded 1 level 4) | + |
| BDNF | 2 | 54 (27/27) | Downgraded 1 level 1) | Downgraded 1 level 2) | Not downgraded | Downgraded 1 level 3) | Downgraded 1 level 4) | + |

Note: 1) There are biases in random methods, allocation concealment, and blind methods; 2) The number of studies is relatively small; 3) The sample size is too small; 4) High heterogeneity
